# Characterization of Muscle Synergies During Activities of Daily Living Using Surface EMG: A Functional Reference for Neuromuscular Assessment

**DOI:** 10.3390/jcm15135268

**Published:** 2026-07-06

**Authors:** Ana Poveda-García, Elisabet Huertas-Hoyas, Cristina García-Bravo, Jorge Pérez-Corrales, Mª Pilar Rodríguez-Pérez, Elisa Bullón-Benito

**Affiliations:** 1 Department of Physical Therapy, Occupational Therapy, Rehabilitation and Physical Medicine, Universidad Rey Juan Carlos, 28922 Alcorcón, Spain; ana.poveda@urjc.es (A.P.-G.); elisabet.huertas@urjc.es (E.H.-H.); cristina.bravo@urjc.es (C.G.-B.); elisa.bullon@urjc.es (E.B.-B.); 2 Research Group Participation, Roles, Occupations and Activities for the Transformation of Communities, Universidad Rey Juan Carlos, 28922 Alcorcón, Spain; 3 Research Group of Humanities and Qualitative Research in Health Science, Department of Physical Therapy, Occupational Therapy, Rehabilitation and Physical Medicine, Universidad Rey Juan Carlos, 28922 Alcorcón, Spain

**Keywords:** muscle synergy, electromyography, central nervous system

## Abstract

**Background/Objectives:** The muscle synergy framework suggests that the central nervous system simplifies hand motor control by recruiting coordinated groups of muscles. However, the organization of these synergies across functional grasp types representative of activities of daily living remains incompletely understood. This study aimed to characterize muscle synergies across functional grasps and identify shared coordination patterns relevant for neuromuscular assessment. **Methods:** Muscle synergies were analysed in 26 healthy participants using a publicly available surface electromyography dataset. Five representative functional grasp types were selected, (cylindrical, lateral pinch, lumbrical, oblique, and tridigital pinch) and synergies were extracted using non-negative matrix factorization. **Results:** Four to five muscle synergies accounted for more than 90% of EMG variance across all grasp types. Despite grasp-specific differences, a consistent set of shared synergies was identified across conditions, explaining 92.5% of the total variance and being flexibly modulated depending on task demands. Extensor-related components showed a particularly consistent contribution across grasps. **Conclusions:** Functional hand grasping relies on a compact and reusable set of muscle synergies that are flexibly adapted to task demands. These findings support a modular organization of neuromuscular control and provide normative references that may be useful for the assessment of altered motor control in neuromuscular disorders, with potential applications in neurorehabilitation and assistive technologies.

## 1. Introduction

The human hand is an extraordinarily complex biomechanical system that enables the execution of a wide range of activities of daily living (ADLs), from power grasps to highly dexterous precision manipulations. With 27 degrees of freedom distributed across 15 joints and controlled by more than 30 muscles, the hand represents one of the most complex effector systems in the human body [1,2,3]. This high dimensionality poses a substantial challenge for the central nervous system (CNS), which must coordinate multiple muscles in real time to produce smooth, accurate, and adaptable movements.

Understanding how the CNS manages this complexity is essential for explaining the neural control of functional hand movements that underpin everyday activities. Moreover, this knowledge has direct implications for neurorehabilitation, the development of myoelectric prostheses, and the design of human–machine interfaces, where efficient and biologically plausible control strategies are required to restore or replicate hand function.

An influential hypothesis originally proposed by Bernstein suggests that the CNS reduces the dimensionality of motor control by organizing muscles into functional units known as muscle synergies [1,4]. Rather than controlling each muscle independently, coordinated groups of muscles may be activated, reducing the number of control variables while preserving functional flexibility [5,6,7]. This synergy-based control strategy has been documented across a wide range of motor behaviors, including locomotion, posture, reaching, and grasping [7].

Within the context of hand motor control, electromyography (EMG)-based studies have shown that muscle activity during grasping can be decomposed into a small number of muscle synergies using dimensionality reduction techniques such as non-negative matrix factorization (NMF) [8,9]. These synergies are typically characterized by time-invariant muscle weightings and task-dependent activation coefficients [6,9], and have been shown to be partially shared across individuals, tasks, and grasp configurations [10,11,12].

Consistently, between three and five muscle synergies have been reported to account for more than 80–90% of the variance in hand and forearm muscle activity during grasping [8,10,12]. Functionally, these synergies have been associated with wrist stabilization, finger flexion–extension patterns, and thumb-related actions, supporting their role as flexible building blocks of grasp control [13,14].

Despite this extensive literature, most studies have focused on a limited set of grasp configurations or highly constrained experimental tasks. Consequently, fewer investigations have systematically examined how muscle synergies are organized and shared across multiple functional grasp types representative of real-world ADLs.

In this context, the present study aims to investigate the muscle synergies involved in five functional grasp types using EMG data from a large public dataset [14]. By analysing cylindrical, lateral pinch, lumbrical, oblique, and tridigital (corresponding to the intermediate grasp defined by Roda et al. [14]) grasps, hand configurations defined according to Roda et al. [14] are analysed to explore the organization of hand muscle synergies during activities of daily living, identifying both shared and task-specific coordination patterns associated with neuromuscular control strategies.

Specifically, the study (i) identifies and characterizes muscle synergies associated with each grasp type by quantifying the contribution of wrist, finger, and thumb muscle groups; (ii) compares synergy structures across grasp conditions to assess similarities and differences in neuromuscular organization; and (iii) evaluates the presence of shared muscle synergies using a global analysis to identify invariant neural modules recruited across tasks.

Overall, this work proposes a quantitative synergy-based framework to improve understanding of neural control of hand function, with potential applications in neurorehabilitation, prosthetic control, and motor impairment assessment.

## 2. Materials and Methods

### 2.1. Study Design

This study followed a cross-sectional observational design. All analyses were performed using pre-existing data from the ERGOMOVMUS database [14], which includes kinematic and electromyographic recordings collected during the performance of activities of daily living. The use of this dataset was considered appropriate for the study objectives, as it provides standardized and high-quality recordings of representative grasp patterns. Collecting new data was beyond the scope of the present study, and the use of this validated database allowed for a robust analysis of hand muscle synergies.

### 2.2. Participants

The dataset included 26 healthy participants (13 males and 13 females; age: 25.5 ± 3.8 years; hand length: 18.4 ± 1.2 cm; hand breadth: 8.4 ± 0.6 cm). All participants performed a total of 161 activities of daily living involving 105 different objects with varying physical characteristics, including both common household items and assistive devices. Written informed consent was obtained from all participants, and the original data collection protocol was approved by the Ethics Committee of Universitat Jaume I (reference CD/021/2018).

### 2.3. Procedure

Five functional grasp types were selected for analysis: cylindrical, lateral pinch, lumbrical, oblique, and tridigital pinch, chosen due to their prevalence in activities of daily living and biomechanical diversity.

Surface electromyography (SEMG) signals were recorded from seven extrinsic muscle groups of the right forearm using Delsys Trigno wireless sensors at 2000 Hz. Electrode placement followed SENIAM guidelines [15] and established forearm EMG protocols [16]. The selected recording sites were based on the forearm mapping methodology proposed by Jarque-Bou et al. [12], which identified skin zones exhibiting distinct and representative muscle activation patterns during activities of daily living. The recorded muscle groups were: flexor carpi ulnaris (WF_UD, wrist flexion and ulnar deviation), flexor carpi radialis and palmaris longus (WF_RD, wrist flexion and radial deviation), superficial and deep finger flexors together with flexor pollicis longus (FF, finger flexion), abductor pollicis longus and extensor pollicis longus/brevis (TM, thumb extension and abduction/adduction), extensor digitorum communis (FE, finger extension), extensor carpi ulnaris (WE_UD, wrist extension and ulnar deviation), and extensor carpi radialis longus/brevis together with brachioradialis and pronator teres (WE_RD, wrist extension and radial deviation).

All EMG signals were processed using a root mean square (RMS) envelope with a 200 ms moving window and normalized to maximum voluntary contraction (MVC), resulting in amplitude values ranging from 0 to 1 [14]. Signals were organized into a non-negative matrix, where muscles were represented as rows and time samples as columns, and used as input for subsequent muscle synergy extraction.

Temporal normalization was not applied prior to muscle synergy extraction. Within each grasp category, all participants contributed the same number of time samples, resulting in identical task durations for a given grasp type. Consequently, no inter-participant variability in signal length was present within each grasp category that could bias the NMF decomposition.

For the global analysis, EMG data from all grasp types were concatenated without temporal normalization, preserving the original temporal structure of each recording.

Muscle synergies were extracted using non-negative matrix factorization (NMF), implemented in Python 3.10 using the scikit-learn library. Synergy vectors were normalized to enable comparison across conditions.

Two complementary analyses were performed: (i) a grasp-specific analysis, in which EMG data were decomposed separately for each grasp type, and (ii) a global analysis, in which EMG data from all grasp types were concatenated to extract shared synergies across conditions.

### 2.4. Statistical Analysis

The number of synergies was determined using the Variance Accounted For (VAF) criterion, which quantifies the percentage of EMG signal variance reconstructed by the NMF model. NMF was iteratively applied varying the number of synergies from 1 to 7, and the minimum number explaining at least 90% of the variance was selected as the optimal solution, following commonly used approaches in muscle synergy analysis [6,13,17].

Descriptive statistics (mean ± standard deviation) were computed for synergy weights and variance explained across grasp types. Synergy structures were visualized using heatmaps and boxplots to illustrate muscle contribution patterns and variability across conditions. Differences in synergy activation coefficients across grasp types were assessed using Kruskal–Wallis H tests (*p* < 0.05).

All analyses were conducted using Python 3.10 with NumPy, pandas, scikit-learn, matplotlib, and seaborn.

## 3. Results

### 3.1. Grasp-Specific Muscle Synergies

#### 3.1.1. Number of Synergies and Variance Explained

**Figure 1:**
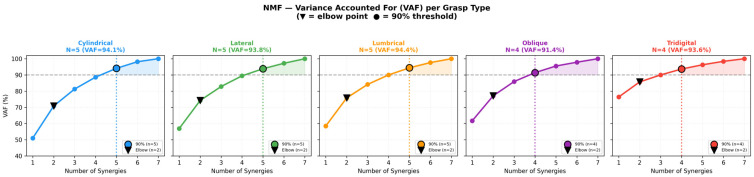
VAF curves as a function of the number of NMF synergies for each of the five grasp types. The dashed horizontal line indicates the 90% VAF threshold. Filled circles mark the selected optimal synergy number based on the 90% VAF criterion. Black triangles indicate the elbow point estimated using the kneedle algorithm. Shaded areas highlight the region above the threshold.

The Cylindrical, Lateral, and Lumbrical grasps required five muscle synergies to explain more than 90% of the EMG variance, whereas the Oblique and Tridigital pinch grasps were adequately represented by four synergies (Figure 1).

All grasp types exceeded the predefined 90% VAF threshold, with values ranging from 91.41% for the Oblique grasp to 94.39% for the Lumbrical grasp (Table 1). Elbow point analysis further indicated a consistent inflection at *n* = 2 synergies across all grasp types, as identified using the kneedle algorithm, although this solution accounted for only 70–86% of the total variance, highlighting the trade-off between model parsimony and reconstruction accuracy.

#### 3.1.2. Synergy Structure

This section describes the structural organization of muscle synergies, focusing on the relative contribution (weights) of each muscle to the extracted synergy modules across grasp types.

**Figure 2:**
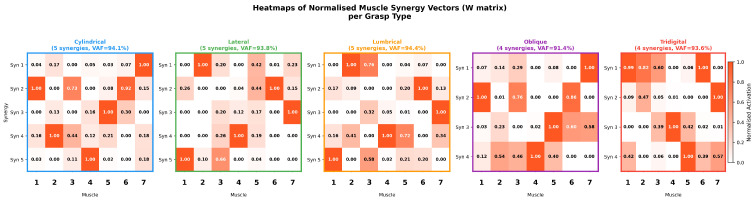
Heatmaps of normalised muscle synergy vectors (W matrix) for each grasp type. Rows represent individual synergies; columns represent the seven extrinsic hand muscles: (1) flexor carpi ulnaris (WF_UD), (2) flexor carpi radialis and palmaris longus (WF_RD), (3) superficial and deep finger flexors together with flexor pollicis longus (FF), (4) abductor pollicis longus and extensor pollicis longus/brevis (TM), (5) extensor digitorum communis (FE), (6) extensor carpi ulnaris, and (7) extensor carpi radialis longus/brevis together with brachioradialis and pronator teres. Colour intensity reflects the normalised contribution of each muscle to the synergy (0 = no contribution, 1 = maximum contribution).

The synergy vectors extracted from the W matrix, representing the relative contribution of each of the seven extrinsic hand muscles to each synergy (Figure 2). The resulting heatmaps illustrate distinct patterns of muscle weighting across synergies and grasp types.

Across all conditions, the synergies exhibit sparse and functionally specific activation patterns, where different muscle groups contribute differentially to each synergy module. These weight distributions define the structural organization of the muscle synergies underlying each grasp type.

Clear differences in synergy structure are observed across grasp types. In the cylindrical grasp, which is characterised by high force generation and global hand stability, several synergies show distributed contributions across wrist flexor and extensor muscles. In particular, the flexor carpi ulnaris, flexor carpi radialis together with palmaris longus, and wrist extensor muscles contribute substantially across different synergies, reflecting the need for coordinated flexion–extension control to stabilise the object.

In the lateral pinch, associated with precision tasks requiring thumb control, the synergies exhibit more selective activation patterns, with a prominent contribution from thumb-related muscles (abductor pollicis longus and extensor pollicis longus/brevis) and wrist stabilisers. Some synergies show minimal contribution from the remaining muscles, indicating more focal and task-specific recruitment.

In the lumbrical grasp, which involves intermediate finger configurations, the synergy structure shows a more balanced distribution of activation across finger flexor muscles and wrist stabilisers. This suggests a more homogeneous coordination of forearm musculature, combining both fine finger control and proximal stabilisation.

In the oblique grasp, a reduced number of synergies is required and the resulting structure is more compact. In this condition, wrist extensor muscles (extensor carpi ulnaris and extensor carpi radialis longus/brevis) show a dominant contribution in several synergies, suggesting an increased role of proximal stabilisation.

In the tridigital pinch, associated with low-force precision tasks, synergies exhibit lower overall activation magnitude. The contribution is mainly concentrated in finger flexor muscles, while proximal forearm muscles show reduced involvement, consistent with the fine and controlled nature of the task.

Overall, the results indicate that each grasp type can be described using a small set of sparse muscle synergies with clearly differentiated contributions from the recorded forearm muscles, reflecting a modular organisation of neuromuscular control of the hand.

#### 3.1.3. Synergy Activation Patterns

**Figure 3:**
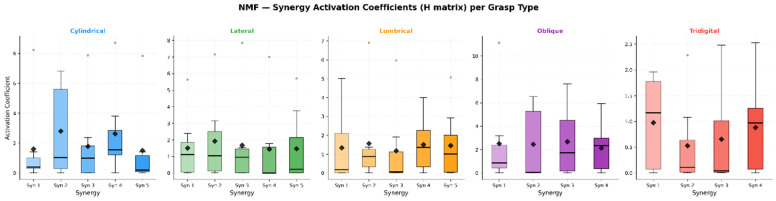
Boxplots of synergy activation coefficients (H matrix) for each grasp type and synergy. Diamond markers indicate mean values. Outliers represent high-demand task conditions.

This section reports the activation patterns of the extracted muscle synergies, focusing on their recruitment magnitude and variability across grasp types (Figure 3).

Synergy activation coefficients showed clear differences in magnitude and variability across grasp types, reflecting task-dependent modulation of muscle synergies.

The cylindrical grasp exhibited relatively high activation across all synergies, with Syn2 (2.80 ± 2.84), Syn4 (2.63 ± 2.70), and Syn3 (1.79 ± 2.61) showing the highest mean values. The lateral grasp presented intermediate activation levels with a more homogeneous distribution across synergies, with mean values ranging between 1.45 and 1.93.

The lumbrical grasp showed moderate activation with a slightly more balanced contribution across synergies, particularly Syn2 (1.56 ± 2.24) and Syn4 (1.51 ± 1.39). The oblique grasp exhibited the highest overall activation magnitudes, especially in Syn3 (2.67 ± 2.72) and Syn1 (2.51 ± 3.66), indicating increased synergy recruitment demands during this condition.

In contrast, the tridigital pinch showed the lowest activation levels across all synergies, with mean values below 1 in most cases (e.g., Syn2: 0.53 ± 0.80), reflecting reduced global muscle recruitment during fine precision tasks (Table 2).

Overall, these results indicate that synergy activation is higher and more variable in grasp types requiring greater force and proximal stabilisation, whereas precision grasps rely on lower and more focal activation patterns. This suggests a task-dependent scaling of synergy recruitment rather than a change in the underlying synergy structure.

Statistical comparisons using Kruskal–Wallis tests confirmed that there were no significant differences in synergy activation coefficients across grasp types for any synergy (all *p* > 0.84), supporting this interpretation.

### 3.2. Global Muscle Synergy Organization

**Figure 4:**
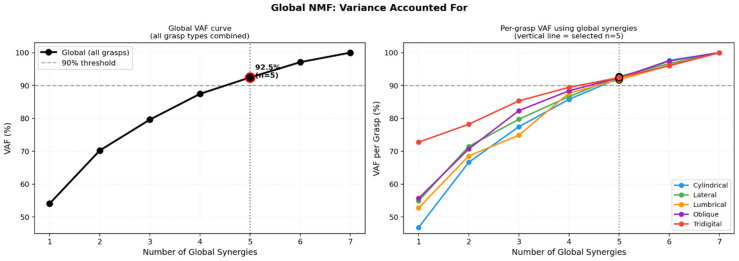
Variance Accounted For (VAF) of the global NMF model. (**Left**) VAF curve obtained by progressively increasing the number of global synergies in the combined analysis of all grasp types, indicating the selection of five synergies as the optimal solution (VAF = 92.5%). (**Right**) VAF by grasp type using the global synergy set, showing that with five synergies the 90% explained variance threshold is exceeded for all grasp types.

A global NMF was performed by concatenating all grasp types into a single dataset (Figure 4). The global model identified five muscle synergies, explaining 92.5% of total variance.

When applied back to each grasp type, this global structure exceeded the 90% VAF threshold in all cases, confirming the existence of a shared low-dimensional structure across tasks.

#### Global Synergy Structure

**Figure 5:**
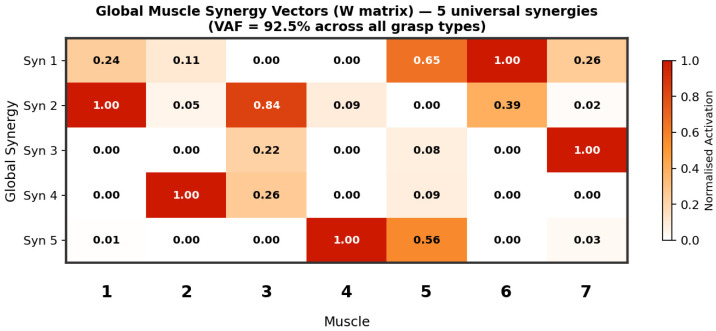
Global muscle synergy vectors (W matrix). Heatmaps of the global muscle synergy vectors (W matrix) obtained from a joint NMF analysis across all grasp types. Five universal synergies were identified, jointly explaining 92.5% of the total variance (VAF). Columns represent the seven extrinsic hand muscles: (1) flexor carpi ulnaris, (2) flexor carpi radialis and palmaris longus, (3) superficial and deep finger flexors together with flexor pollicis longus, (4) abductor pollicis longus and extensor pollicis longus/brevis, (5) extensor digitorum communis, (6) extensor carpi ulnaris, and (7) extensor carpi radialis longus/brevis together with brachioradialis and pronator teres. Color intensity indicates the normalized contribution of each muscle to each synergy (0 = no contribution; 1 = maximum contribution).

**Figure 6:**
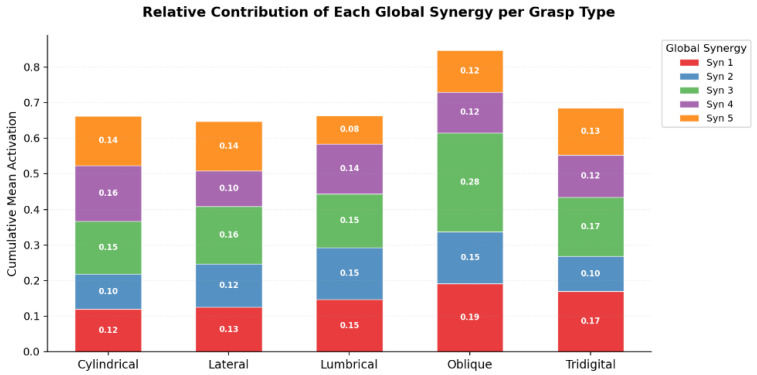
Relative contribution of each global synergy by grasp type. Mean relative contribution of each of the five global muscle synergies across the different grasp types (Cylindrical, Lateral, Lumbrical, Oblique, and Tridigital). Each bar represents the cumulative mean activation of the global synergies for each grasp, illustrating the relative weight of each synergy within the overall pattern of muscle coordination.

The global W matrix (Figure 5 and Figure 6) revealed five consistent muscle synergies that jointly explained 92.5% of the total variance. Each synergy showed a distinct pattern of muscle contributions. Syn 1 was predominantly associated with the extensor carpi ulnaris and extensor digitorum communis, while Syn 2 was mainly driven by the flexor carpi ulnaris together with the finger flexors, including flexor digitorum superficialis, flexor digitorum profundus, and flexor pollicis longus. Syn 3 was characterized by a strong contribution from extensor carpi radialis longus and brevis, whereas Syn 4 was dominated by flexor carpi radialis and palmaris longus, with an additional contribution from the finger flexors. Finally, Syn 5 was primarily associated with abductor pollicis longus and extensor pollicis longus and brevis, with a secondary contribution from extensor digitorum communis.

Across all synergies, wrist and finger muscles were consistently represented, indicating coordinated activation patterns. The relative contribution of each synergy varied across grasp types, as illustrated in Figure 6.

**Figure 7:**
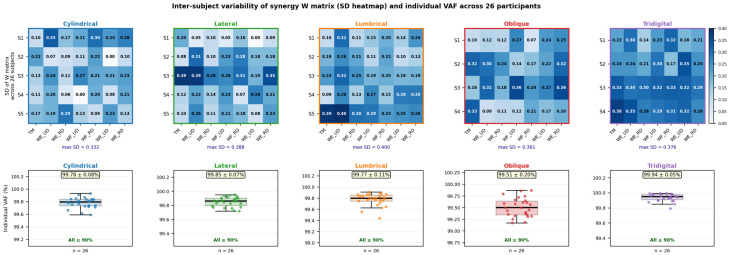
Inter-subject variability of muscle synergy vectors and individual reconstruction quality across 26 participants. (**Top row**) Standard deviation (SD) of normalised synergy weight vectors (W matrix) across the 26 participants for each grasp type. Cell values indicate the SD of each muscle weight within each synergy (S1–S5 or S1–S4); darker shading reflects greater inter-individual variability. (**Bottom row**) Individual variance accounted for (VAF, %) for each participant when their EMG data were reconstructed using the group-level synergies. Each dot represents one participant; the box shows the interquartile range and median. All 26 participants exceeded the 90% VAF threshold for every grasp type, confirming that the pooled synergies are representative of individual muscle coordination patterns.

To further assess whether the pooled synergies were representative of individual participants, a subject-level validation analysis was performed. All participants showed consistently high VAF values across grasp types (Figure 7), indicating that the extracted synergies adequately reconstruct individual EMG patterns and supporting the validity of the pooled approach.

## 4. Discussion

The results of this study suggest that neuromuscular coordination of the hand during functional grasping can be effectively described using a reduced set of muscle synergies, both in task-specific and global analyses. Importantly, statistical analyses confirmed that the observed differences in synergy activation patterns across grasp types did not reach statistical significance. This indicates that although descriptive differences in activation magnitude can be observed between grasp types, the underlying modulation does not reflect strong or discrete separations between conditions. Instead, grasp-related variations appear to emerge from subtle changes in the weighting and recruitment of a largely shared synergy set, rather than from fundamentally distinct activation regimes. This further supports the interpretation that hand control is organised around a stable low-dimensional set of synergies that is flexibly modulated across functional tasks.

Compared to previous studies such as Castellini et al. [8] and Gracia-Ibáñez et al. [10], which primarily focused on muscle synergy extraction under more limited or task-specific experimental conditions, the present study extends this work by integrating both task-specific and global synergy analyses across a broader set of functional grasp types within a unified framework. This allows a more comprehensive characterization of hand motor coordination, capturing both task-dependent modulation and shared synergy structures across activities of daily living.

These findings indicate that all grasp types can be effectively described using a low-dimensional synergy structure, with minimal differences in variance explained across conditions. This reduction in dimensionality is consistent with motor control theories proposing that complex motor behaviour emerges from the flexible combination of a limited set of coordinative modules rather than independent muscle control [17,18] and is aligned with the original concept of motor redundancy reduction proposed by Bernstein [1] and further developed in synergy-based frameworks [19].

In the task-specific analyses, four to five muscle synergies were sufficient to explain more than 90% of EMG variance across all grasp types. This finding aligns with previous literature reporting similar dimensionality during grasping and manipulation tasks under different experimental conditions [20,21,22] and is consistent with EMG-based synergy studies showing robust low-dimensional representations across subjects and tasks [10,12]. Rather than reflecting discrete functional categories, differences between grasp types appear to arise from continuous variations in the relative weighting of shared synergies shaped by biomechanical constraints.

A consistent finding of this study across all grasp types was the involvement of specific muscles across multiple synergies, particularly extensor digitorum communis and wrist extensor groups, which acted as dominant muscles in some synergies and contributed to others, reflecting their participation in different combinations of muscle activation across tasks. This suggests that extensor musculature plays an active and structural role in grasp coordination, contributing not only to joint stabilization but also to the regulation of global hand stiffness and coordinated movement patterns. Rather than acting as passive antagonists to flexors, these muscles appear to be integral components of functional motor modules [23,24]. This observation is consistent with neurophysiological evidence highlighting flexor–extensor coactivation as a fundamental mechanism for ensuring stability and precision during object manipulation [19,25]. However, it should be noted that surface EMG does not allow a clear distinction between muscle activation related to active force generation and co-contraction for joint stabilization. Therefore, these interpretations should be considered in terms of overall muscle activation rather than specific functional roles.

Similarly, the recurrent presence of synergies dominated by wrist extension suggests that proximal stabilization of the hand constitutes a shared and reusable control component across diverse functional grasps. The consistent involvement of the thumb further reinforces its central role in grasp configuration and object interaction, in particular, abductor pollicis longus and extensor pollicis longus and brevis showed contributions across different synergies, consistent with their functional relevance in manual dexterity [3,18,26]. These findings are also consistent with kinematic studies showing that hand posture and digit coordination can be described using low-dimensional structures shared across tasks and individuals [10,11,12].

The global NMF analysis revealed that a small set of shared muscle synergies is sufficient to reconstruct EMG activity across all grasp types. Importantly, the correspondence between global and task-specific synergies suggests that functional specificity emerges primarily through modulation of synergy activation coefficients rather than changes in synergy structure [17,27]. This supports the hypothesis that muscle synergies represent reusable neural modules that are flexibly combined across contexts [7,17,18] and is consistent with optimal and predictive control frameworks [28,29,30].

An additional analysis of the activation coefficients (H matrix) at the task level provides further insight into the observed variability across grasp types. The variability in synergy activation across activities of daily living reflects systematic differences in functional demands within each grasp category. Specifically, tasks involving higher force or precision requirements (e.g., opening a jar or using a hammer in cylindrical grasps) elicited higher synergy activation levels, whereas simpler actions such as holding a cup or grasping lightweight objects were associated with lower activation values. These findings indicate that the observed variability is not due to noise or methodological artifacts, but rather to task-dependent modulation of synergy recruitment. In this context, muscle synergies appear to be flexibly scaled according to the biomechanical requirements of each activity while preserving a stable underlying synergy structure.

From an applied perspective, the identification of stable and shared muscle synergies has relevant implications for rehabilitation, neuroengineering, and prosthetic control. Low-dimensional synergy-based representations may simplify control strategies while preserving functional richness, offering a biologically grounded framework for intuitive human–machine interaction [31,32]. In prosthetic applications, these representations could enable more natural and adaptive multi-grasp control by mapping neural intent to coordinated muscle activations, reducing user cognitive load. In clinical contexts, synergy analysis also provides a quantitative tool to characterise neuromuscular alterations and monitor functional recovery, particularly in neurorehabilitation settings [17,33,34]. Additionally, in neuroengineering, synergy-based models may support the development of more robust decoding algorithms for brain–machine interfaces by constraining control to physiologically meaningful motor modules.

Overall, these findings support a model of hand motor control based on the flexible recruitment of a limited set of shared muscle synergies. This modular organization enables a wide variety of functional grasps to emerge from a low-dimensional control space, with task-dependent behaviour primarily driven by modulation of synergy activation rather than structural reconfiguration. This framework reconciles the apparent complexity of hand biomechanics with an efficient neural control strategy and provides a coherent basis for understanding dexterous manipulation in daily life [17,18].

Several limitations of this study should be acknowledged. First, the analysis was based exclusively on surface electromyography (EMG) recordings from seven extrinsic hand muscles, which does not capture the contribution of intrinsic hand muscles. These muscles play a key role in fine motor control and finger individuation, and their absence may have led to an incomplete characterization of the full neuromuscular synergy structure underlying precision grasping. In addition, intrinsic muscles contribute substantially to dexterous object manipulation and force modulation, aspects that may not be fully represented by the extrinsic musculature alone.

Second, the use of surface EMG inherently limits the spatial specificity of muscle activity due to potential cross-talk between adjacent muscles and the inability to directly measure deep muscle activity. Consequently, some recorded activation patterns may reflect overlapping contributions from neighboring muscles, potentially influencing synergy extraction and interpretation.

Third, the EMG signals were processed using a root mean square (RMS) window of 200 ms, as defined in the original publicly available dataset used in this study. Consequently, this preprocessing parameter could not be modified. Although this window provides a stable estimate of muscle activation, it may smooth short-lived EMG fluctuations, potentially reducing sensitivity to rapid changes in muscle synergy activation during dynamic grasp adjustments.

Fourth, the study focused on a predefined set of functional grasp types extracted from an existing dataset of activities of daily living. While this increases ecological validity, it also introduces variability in task execution conditions that may influence synergy estimation. Future studies using more controlled experimental protocols could help isolate task-specific effects with greater precision. Furthermore, although the selected tasks were representative of activities of daily living, they were performed within an experimental framework and therefore may not fully capture the spontaneity, variability, and adaptive characteristics of hand movements in real-world environments. As a result, the identified synergies should be interpreted as representative of the selected task repertoire rather than an exhaustive description of natural hand function.

Fifth, the participant sample consisted exclusively of young healthy individuals. While this provides a valuable normative reference for understanding hand muscle coordination, caution is warranted when extrapolating these findings to older adults or clinical populations, whose neuromuscular control strategies may differ substantially due to aging, injury, or neurological impairment.

Additionally, the present analysis is cross-sectional and does not address how muscle synergies may evolve over time, adapt with learning, or change in response to fatigue or pathology. Longitudinal studies would be necessary to understand the stability and plasticity of the identified synergy structures.

Future research should extend this work by including intrinsic muscle recordings using high-density surface EMG or intramuscular techniques, which would allow a more complete characterization of hand muscle coordination. Additionally, time-resolved or dynamic synergy analysis could be applied to investigate how muscle synergies evolve during the full temporal sequence of grasping and object manipulation.

This study used an existing publicly available dataset of electromyographic recordings rather than collecting new experimental data. The dataset provides high-quality and well-standardised EMG measurements across a wide range of functional grasping tasks in activities of daily living, with all recordings acquired under a controlled protocol, standardised sensor placement, and synchronised task execution. The use of this dataset ensures strong experimental consistency across subjects and conditions, which is particularly relevant for muscle synergy analysis, where comparability across tasks is essential. In addition, the availability of a pre-processed and well-characterised dataset allows for reproducible analysis and facilitates direct comparison across different grasping conditions within a unified experimental framework.

In clinical contexts, muscle synergy analysis has been increasingly used to characterize neuromuscular impairment following neurological injury. In post-stroke individuals, recent evidence shows that synergies may exhibit reduced dimensionality and altered structure, including merging or fractionation, particularly in more severely affected patients, suggesting a reorganization of motor control strategies and a relationship with impairment severity [34]. These alterations have been proposed as potential biomarkers of motor dysfunction, reflecting changes in intermuscular coordination and reduced independent joint control.

In addition, recent rehabilitation studies indicate that changes in synergy structure and activation patterns may be associated with functional recovery, supporting their use for tracking motor improvement over time [35]. However, the literature also highlights substantial methodological heterogeneity across studies, including differences in EMG processing and synergy extraction methods, which limits direct clinical translation and comparability of findings [34]. Therefore, while muscle synergy analysis shows potential to characterize neuromuscular coordination in stroke and may support future individualized rehabilitation strategies, its role in guiding specific clinical decision-making remains exploratory. Similarly, integration of muscle synergy models into prosthetic control systems has the potential to improve the naturalness and efficiency of human–machine interaction by exploiting low-dimensional control strategies.

Overall, these findings support a framework of hand motor control based on the flexible recruitment of a limited set of shared muscle synergies, providing a low-dimensional representation of complex hand movements and a coherent basis for understanding dexterous manipulation in daily activities.

## 5. Conclusions

This study suggests that functional hand grasping during activities of daily living can be described using a small set of muscle synergies that explain most of the EMG variance, supporting a low-dimensional organization of neuromuscular control. Across all grasp types, a consistent set of shared synergies was identified and flexibly modulated through task-dependent activation rather than structural reorganization. Wrist and finger extensor muscles showed a consistently strong contribution, highlighting their central role in grasp stabilization and coordinated hand function.

Overall, these findings support the concept of a modular and reusable organization of motor control and provide normative insights into physiological muscle coordination during functional hand tasks. The identified synergy patterns may contribute to the development of objective EMG-based tools for the assessment of altered motor control in neuromuscular disorders, as well as to future applications in neurorehabilitation, functional evaluation, and assistive or prosthetic technologies.

## Figures and Tables

**Table 1:** NMF results: optimal number of synergies and VAF per grasp type.

Grasp Type	N Observations	Optimal Synergies	VAF (%)
Cylindrical	2595	5	94.08
Lateral pinch	1508	5	93.80
Lumbrical	987	5	94.39
Oblique	1508	4	91.41
Tridigital pinch	156	4	93.64

**Table 2:** Grasp-specific muscle synergy activation (H matrix) statistics and representative ADL task modulation. Mean ± SD and median of muscle synergy activation coefficients (H matrix) for each grasp type. Syn 1–5 represent muscle synergies extracted using non-negative matrix factorization (NMF). The table also reports representative activities of daily living (ADL) tasks associated with the highest (↑) and lowest (↓) activation coefficients for each synergy, providing task-level context for the observed variability.

Grasp	Metric	Syn1	Syn2	Syn3	Syn4	Syn5
Cylindrical	H value (Mean ± SD/Median)	1.60 ± 2.74/ 0.40	2.80 ± 2.84/ 1.04	1.79 ± 2.61/ 0.99	2.63 ± 2.70/ 1.55	1.50 ± 2.63/ 0.20
	↑ Highest activation tasks	Shelf transport	Horizontal transport, pouring	Shelf transport	Shelf transport	Pouring, horizontal transport
	↓ Lowest activation tasks	Hairbrush, tap, spray	Door handle	Door handle, hairbrush	Hairbrush, light pouring	Light shelf transport
Lateral	H value (Mean ± SD/Median)	1.51 ± 1.87/1.12	1.93 ± 2.39/1.04	1.67 ± 2.60/0.95	1.45 ± 2.37/0.00	1.46 ± 2.14/0.23
	↑ Highest activation tasks	Screw cap, pouring	Pouring, shelf transport	Plug in/out, pouring	Pouring, screw cap	Shelf transport, screw cap, pouring
	↓ Lowest activation tasks	Light shelf transport	Connector push/pull	Light pouring, shelf	Connector push/pull	Plug in/out
Lumbrical	H value (Mean ± SD/Median)	1.34 ± 1.97/ 0.18	1.56 ± 2.24 / 0.87	1.18 ± 2.06/ 0.05	1.51 ± 1.39/ 1.36	1.45 ± 1.76/ 1.01
	↑ Highest activation tasks	Shelf transport (heavy)	Shelf transport (heavy)	Shelf + pouring (heavy)	Shelf transport (heavy)	Pouring (heavy)
	↓ Lowest activation tasks	Pouring (light)	Shelf transport (light)	Pouring (light)	Pouring (light)	Pouring (light)
Oblique	H value (Mean ± SD/Median)	2.51 ± 3.66/ 0.86	2.45 ± 2.84/ 0.06	2.67 ± 2.72/ 1.72	2.14 ± 1.96/ 2.35	—
	↑ Highest activation tasks	Shelf transport	Pouring, shelf transport	Shelf transport	Screwdriver, iron (torque)	—
	↓ Lowest activation tasks	Screwdriver, hairbrush	Hairbrush, screwdriver	Hairbrush, light pouring	Shelf transport	—
Tridigital	H value (Mean ± SD/Median)	0.98 ± 0.84/ 1.17	0.53 ± 0.80/ 0.11	0.66 ± 0.86/ 0.04	0.88 ± 0.86/ 0.97	—
	↑ Highest activation tasks	Pen/writing	Pen/writing	Pen/writing	Pen/writing	—
	↓ Lowest activation tasks	Lipstick	Lipstick	Lipstick	Lipstick	—

## Data Availability

Publicly available datasets were analyzed in this study. The data are available in the ERGOMOVMUS (MOVMUS-UJI) database described by Roda-Sales et al. [14] ERGOMOVMUS (MOVMUS-UJI) and deposited in the Zenodo repository. No new data were created or analyzed beyond those available in the original dataset.

## Abbreviations

ADLs	activities of daily living
CNS	central nervous system
DF	digit flexion
EMG	electromyography
FE	finger extension
MVC	maximum voluntary contraction
NMF	non-negative matrix factorization
RMS	root mean square
TM	thumb extension/abduction–adduction
VAF	variance accounted
WE_RD	wrist extension + radial deviation
WE_UD	wrist extension + ulnar deviation
WF_RD	wrist flexion + radial deviation
WF_UD	wrist flexion + ulnar deviation

## References

[1] Bernstein N.A. (1967). The Co-Ordination and Regulation of Movements.

[2] Kutch J.J., Valero-Cuevas F.J. (2012). Challenges and new approaches to proving the existence of muscle synergies of neural origin. PLoS Comput. Biol..

[3] Ingram J.N., Körding K.P., Howard I.S., Wolpert D.M. (2008). The statistics of natural hand movements. Exp. Brain Res..

[4] Bizzi E., Cheung V.C. (2013). The neural origin of muscle synergies. Front. Comput. Neurosci..

[5] d’Avella A., Lacquaniti F. (2013). Control of reaching movements by muscle synergy combinations. Front. Comput. Neurosci..

[6] Tresch M.C., Cheung V.C., d’Avella A. (2006). Matrix factorization algorithms for the identification of muscle synergies: Evaluation on simulated and experimental data sets. J. Neurophysiol..

[7] Bizzi E., Cheung V.C., d’Avella A., Saltiel P., Tresch M. (2008). Combining modules for movement. Brain Res. Rev..

[8] Castellini C., van der Smagt P. (2013). Evidence of muscle synergies during human grasping. Biol. Cybern..

[9] Tagliabue M., Ciancio A.L., Brochier T., Eskiizmirliler S., Maier M.A. (2015). Differences between kinematic synergies and muscle synergies during two-digit grasping. Front. Hum. Neurosci..

[10] Gracia-Ibáñez V., Sancho-Bru J.L., Vergara M., Jarque-Bou N.J. (2020). Sharing of hand kinematic synergies across subjects in daily living activities. Sci. Rep..

[11] Jarque-Bou N., Gracia-Ibáñez V., Sancho-Bru J.L., Vergara M., Pérez-González A., Andrés F.J. (2016). Using kinematic reduction for studying grasping postures. An application to power and precision grasp of cylinders. Appl. Ergon..

[12] Jarque-Bou N.J., Vergara M., Sancho-Bru J.L., Gracia-Ibáñez V., Roda-Sales A. (2020). Hand kinematics characterization while performing activities of daily living through kinematics reduction. IEEE Trans. Neural Syst. Rehabil. Eng..

[13] Zhao K., Zhang Z., Wen H., Liu B., Li J., d’Avella A., Scano A. (2023). Muscle synergies for evaluating upper limb in clinical applications: A systematic review. Heliyon.

[14] Roda-Sales A., Jarque-Bou N.J., Bayarri-Porcar V., Gracia-Ibáñez V., Sancho-Bru J.L., Vergara M. (2023). Electromyography and kinematics data of the hand in activities of daily living with special interest for ergonomics. Sci. Data.

[15] Hermens H.J., Freriks B., Disselhorst-Klug C., Rau G. (1999). European Recommendations for Surface Electromyography (SENIAM).

[16] Farina D., Pozzo M., Merlo E., Bottin A., Merletti R. (2004). Assessment of average muscle fiber conduction velocity from surface EMG signals during fatiguing dynamic contractions. IEEE Trans. Biomed. Eng..

[17] Cheung V.C., Turolla A., Agostini M., Silvoni S., Bennis C., Kasi P., Paganoni S., Bonato P., Bizzi E. (2012). Muscle synergy patterns as physiological markers of motor cortical damage. Proc. Natl. Acad. Sci. USA.

[18] Santello M., Baud-Bovy G., Jörntell H. (2013). Neural bases of hand synergies. Front. Comput. Neurosci..

[19] Overduin S.A., d’Avella A., Carmena J.M., Bizzi E. (2014). Muscle synergies evoked by microstimulation are preferentially encoded during behavior. Front. Comput. Neurosci..

[20] Torres-Oviedo G., Macpherson J.M., Ting L.H. (2006). Muscle synergy organization is robust across a variety of postural perturbations. J. Neurophysiol..

[21] Alnajjar F., Wojtara T., Kimura H., Shimoda S. (2013). Muscle synergy space: Learning model to create an optimal muscle synergy. Front. Comput. Neurosci..

[22] Delis I., Panzeri S., Pozzo T., Berret B. (2014). A unifying model of concurrent spatial and temporal modularity in muscle activity. J. Neurophysiol..

[23] Takei T., Seki K. (2010). Spinal interneurons facilitate coactivation of hand muscles during a precision grip task in monkeys. J. Neurosci..

[24] Seo G., Park J.H., Park H.S., Roh J. (2023). Developing new intermuscular coordination patterns through an electromyographic signal-guided training in the upper extremity. J. Neuroeng. Rehabil..

[25] Latash M.L. (2021). Understanding synergy: A single concept at different levels of analysis?. Front. Syst. Neurosci..

[26] Schieber M.H. (1991). Individuated finger movements of rhesus monkeys: A means of quantifying the independence of the digits. J. Neurophysiol..

[27] Vinjamuri R., Patel V., Powell M., Zhi-Hong M., Nathan C. (2014). Candidates for synergies: Linear discriminants versus principal components. Comput. Intell. Neurosci..

[28] Scott S.H. (2012). The computational and neural basis of voluntary motor control and planning. Trends Cogn. Sci..

[29] Franklin D.W., Wolpert D.M. (2011). Computational mechanisms of sensorimotor control. Neuron.

[30] Rigoux L., Guigon E. (2012). A model of reward- and effort-based optimal decision making and motor control. PLoS Comput. Biol..

[31] Zandigohar M., Han M., Sharif M., Günay S.Y., Furmanek M.P., Yarossi M., Bonato P., Onal C., Padır T., Erdoğmuş D. (2024). Multimodal fusion of EMG and vision for human grasp intent inference in prosthetic hand control. Front. Robot. AI.

[32] Tanzarella S., Di Domenico D., Forsiuk I., Boccardo N., Chiappalone M., Bartolozzi C., Semprini M. (2024). Arm muscle synergies enhance hand posture prediction in combination with forearm muscle synergies. J. Neural Eng..

[33] Scotto di Luzio F., Cordella F., Bravi M., Santacaterina F., Bressi F., Sterzi S., Zollo L. (2022). Modification of hand muscular synergies in stroke patients after robot-aided rehabilitation. Appl. Sci..

[34] Facciorusso S., Guanziroli E., Brambilla C., Spina S., Giraud M., Tosatti L.M., Santamato A., Molteni F., Scano A. (2024). Muscle synergies in upper limb stroke rehabilitation: A scoping review. Eur. J. Phys. Rehabil. Med..

[35] Kim D., Ko S.H., Han J., Kim Y.T., Kim Y.H., Chang W.H., Shin Y.I. (2024). Evidence of the existence of multiple modules for the stroke-caused flexion synergy from Fugl-Meyer assessment scores. J. Neurophysiol..

